# Breakdown of Kasha’s Rule in a Ubiquitous, Naturally Occurring, Wide Bandgap Aluminosilicate (Feldspar)

**DOI:** 10.1038/s41598-017-17466-z

**Published:** 2018-01-16

**Authors:** Amit Kumar Prasad, Mayank Jain

**Affiliations:** 1 Center for Nuclear Technologies, Technical University of Denmark, DTU Risø Campus, Roskilde, 4000 Denmark; 2 Present Address: Schulich Faculty of Chemistry and Solid State Institute, Technion-Israel Institute of Technology, Haifa, 32000 Israel

## Abstract

Excitation-energy-dependent emission (EDE) is well known from photoluminescence (PL) studies of polar solvents and carbon-based nanostructures. In polar solvents, this effect known as the ‘red edge effect’ (REE) is understood to arise from solute-solvent interactions, whereas, in case of carbon-based nanostructures, the origin is highly debated. Understanding this effect has important bearings on the potential applications of these materials. EDE has never been reported from large crystalline materials, except very recently by our group. Here, we make detailed investigations to understand the universality and the mechanism behind the EDE in a wide band gap aluminosilicate (feldspar), which comprises more than half of the Earth’s crust, and is widely used in geophotonics (e.g., optical dating). We observe EDE up to 150 nm at room temperature in our samples, which is unprecedented in rigid macroscopic structures. Based on PL investigations at 295 K and 7 K, we present a novel model that is based on photoionisation of a deep lying defect and subsequent transport/relaxation of free electrons in the sub-conduction band tail states. Our model has important implications for potential photonic applications using feldspar, measurement of band tail width in wide bandgap materials, and understanding the EDE effect in other materials.

## Introduction

It is well known that fluorescence spectra in viscous polar solvents show a red shift in emission when excitation energy is lowered towards the red edge of the absorption spectrum^1^. This effect known as the red-edge effect (REE) is related to solute-solvent interactions^1–3^. The red-edge effect apparently violates the Kasha’s rule^2,3^, which states that the emission energy is independent of the excitation energy and always occurs from the lowest electronic/vibrational excited state. However, it is now well established that the REE occurs from an ensemble of excited molecules with a distribution of interaction energies with the solvent, thus giving rise to a distribution of emission energies^1^. Over the last four decades since its initial observation^4^, this mechanism has been observed in variety of fluorophores in different solvents ranging from high viscosity solutions, glasses and polymer matrices, etc., characterized by frozen or very slow structural dynamics^1^.

Observations of a similar effect, i.e., a red shift in the emission with a decrease in the excitation energy, has also come from graphene quantum dots and other graphene oxide derivatives. A detailed review on this aspect has been given by Gan *et al*.^5^; these authors note that despite a decade of research, this is still a perplexing phenomenon whose origins are actively debated. Understanding this effect has important implications for development of photonic devices based on nano materials. Various explanations of this effect include size variations, surface defects, and local solute-solvent interactions. For example, a solvent-relaxation induced giant red edge effect has been observed in Graphene Oxide (GO)^6^; this study attributed the giant shift to an additional slow solvation from reorganization of the GO sheet surrounding the localized electron density of the fluorophore. In contrast, the excitation-dependent fluorescence in carbon nanodots has been attributed to multiple discrete electronic states arising from different types of aggregates^7^; this study rules out the role of solvent relaxation and suggests that surface exposed functional groups play a major role in forming such aggregates. Similarly, a strong excitation dependent emission is also observed in poly(ethylene glycol)/carbon quantum dot composite solid films, where a quasi-continuous conduction band (and not the REE) is shown to explain the tunable emission^8^.

Although Excitation (energy) Dependent Emission (henceforth EDE) has perplexed and inspired researchers in the different materials discussed above, viz. viscous, polar solvents, glasses, polymers and carbon based nano materials, it was only recently discovered in a macrocrystalline, wide band-gap material by our group (Prasad *et al.*, 2016)^9^. We reported a large EDE shifts in the steady state PL spectra of a Na-feldspar sample. Based on the exponential distribution of the intensity of the PL emission as a function of excitation energy, we tentatively attributed this effect to the presence of band tail states or a charge transfer band arising from defect clusters; however, neither have these preliminary suggestions been confirmed yet, nor has it been shown that EDE is a universal characteristic of feldspar. Understanding the universality and the origin of EDE in feldspar is particularly important since it is the most common mineral on Earth’s crust (~60% of terrestrial rocks) and other planetary bodies. Feldspar is also widely used in optical dating, where different emissions are used to derive chronologies of the past climatic and archaeological events^10–15^. Furthermore, the EDE, if universal in feldspar or feldspar like materials, has the potential for devising cheap tunable light sources, which is highly attractive given their widespread availability in nature.

The motivation of this study is to explore the universality of the EDE in feldspar, and to understand its origin. It is hoped that this study benefits the application of feldspar in geochronology, as well as gives new physical insights into the mechanism of excitation-energy-dependent emission phenomenon.

## Feldspar: defects, energy structure and charge transport

Feldspar is a crystalline aluminosilicate whose composition can be represented in terms of a ternary system: orthoclase (KAlSi_3_O_8_), albite (NaAlSi_3_O_8_) and anorthite (CaAl_2_Si_2_O_8_). Feldspar crystallises in monoclinic or triclinic systems. It consists of interlinked tetrahedrons of SiO_4_ and AlO_4_ which form a negatively charged 3D framework, and large cations such as K, Na or Ca, which occupy the interstices of the framework^16^.

Feldspar has a wide band gap (E_g_ ~ 7.7 eV), and contains various structural/impurity defects that give rise to energy levels within the band gap^17^. Some of these defects create metastable energy levels by capturing free electrons or holes created by ionizing radiation; this property is exploited in geochronometry (optical dating), an actively developing application in geo-photonics^10–13,18,19^. The use of feldspar as a geochronometer kick started with the discovery of IR stimulated luminescence (IRSL) signal in feldspar^13^; today this signal is widely used to date past geological and archaeological events up to last 0.5 Ma^10–15^. IRSL arises from resonant excitation of electrons in the principal traps using infra-red light (~850 nm), followed by an eventual radiative recombination with trapped holes elsewhere in the lattice^13,19^. The concentration of such trapped electrons in feldspar, buried in sediments or rocks, increases with time due to the impact of the surrounding ionising radiation; thus, the intensity of IRSL signal can eventually be calibrated in terms of sample’s age^10–12^.

Low mobility conduction band tail states play an important role in charge transfer in feldspar. These states arise due to variation in bonding angles, strain, thermal effects and structural disorder in the material^20,21^. Poolton *et al*.^22^ demonstrated that IRSL is generated by transport of photo-excited electrons (~1.45 eV) from the principal trap via diffusion in the band tail states^22^, or by tunnelling from the excited state of the principal trap. Jain and Ankjaergaard^23^ investigated charge transport in the band tail states using the time - resolved Optically Stimulated Luminescence (OSL). They identified two dominant routes within the band tail states depending on the energy of the detrapped electron: a) phonon (0.05–0.06 eV) assisted diffusion, and b) quantum mechanical tunnelling. Based on these observations they presented a comprehensive model of the luminescence generating process in feldspar. According to them, the density of states (DOS) of the band tails and their distribution with energy, is important for understanding the charge transport dynamics in feldspar. In general, the DOS of band tail states have an exponential dependence on energy in semiconductors and insulators^24^:

1 $$\rho (E)\propto \exp (-{E}^{n}/{E}_{b})$$

where *E* is the energy of the state measured away from the band edge and for n = 1 (the Urbach tail) the constant *E*_*b*_ defines the band tail width, i.e., the energy at which the density falls by e^−1^ of that at the band edge^25^.

There have been few attempts to map the band tail states in feldspar. Poolton *et al*.^22^ measured the band tails states using excitation spectroscopy after irradiating the sample with x-rays at cryogenic temperature. They estimated a band tail width of 0.4 eV in a typical Na and K Feldspars. Jain and Ankjærgaard^23^ discussed that the presence of band tail states affects the thermal lifetime of the principal traps in feldspar. Li and Li^26^ developed a mathematical model for analysis of isothermal decay of IRSL signal that includes detrapping to the band tail states following Equation (1) for n = 1. Based on the analysis of isothermal depletion of IRSL and post IR-IRSL, they estimated the band tail width to be ~0.3–0.4 eV. Morthekai *et al*.^27^ analysed time-resolved IRSL data using a variable range hopping mechanism within the band tail states, and concluded that hopping length decreases with stimulation temperature.

The band tail width (E_b_) is an important parameter since it constrains the thermal lifetime of principal traps. This is particularly important for applications in thermocrhonometry using feldspar^28–30^, where it is important to quantify thermally induced electron detrapping probability. However, to date there is no simple and easily available optical method of mapping band tail states in wide band gap materials in general and feldspar in particular. Therefore, parameter E_b_ is indirectly estimated using thermal depletion; this approach has several limitations because of multiple model parameters and their mutual inter-dependence.

## Results: Steady State and Time-Resolved PL Measurements

Steady state PL measurements were carried out on 6 feldspar samples of different composition (see Table 1), and time resolved PL measurements were carried out on R28. Measurements were carried out at room temperature (295 K) or 7 K (see the section: ‘Samples and Experimental Details’). The results are summarised below.

**Table 1:** List of samples, their bulk composition as determined by XRF and Mn content evaluated using ICP-MS.

Sample code	Type of Feldspar	Provenance	Composition on Unity			Mn (ng/g)
			KF	NaF	CaF	
R28	M	Switzerland	0.95	0.05	0	—
R47	S	Tanzania	0.85	0.12	0.03	3.31
R57	M	u/k	0.43	0.53	0.04	48.11
R58	M	u/k	0.27	0.63	0.1	22.3
R64	S	Iceland	0.01	0.15	0.85	155.9
R65	NIST	u/k	0.27	0.73	0	17.47

S represents feldspars of sedimentary origin, and M represents museum single crystal samples. Here, KF: K-Feldspar, NaF: Na-Feldspar, CaF: Ca-Feldspar, u/k: unknown, NIST: A reference feldspar sample from National Institute of Standards Technology.

### Excitation dependence emission (295 and 7 K)

**Figure 1:**
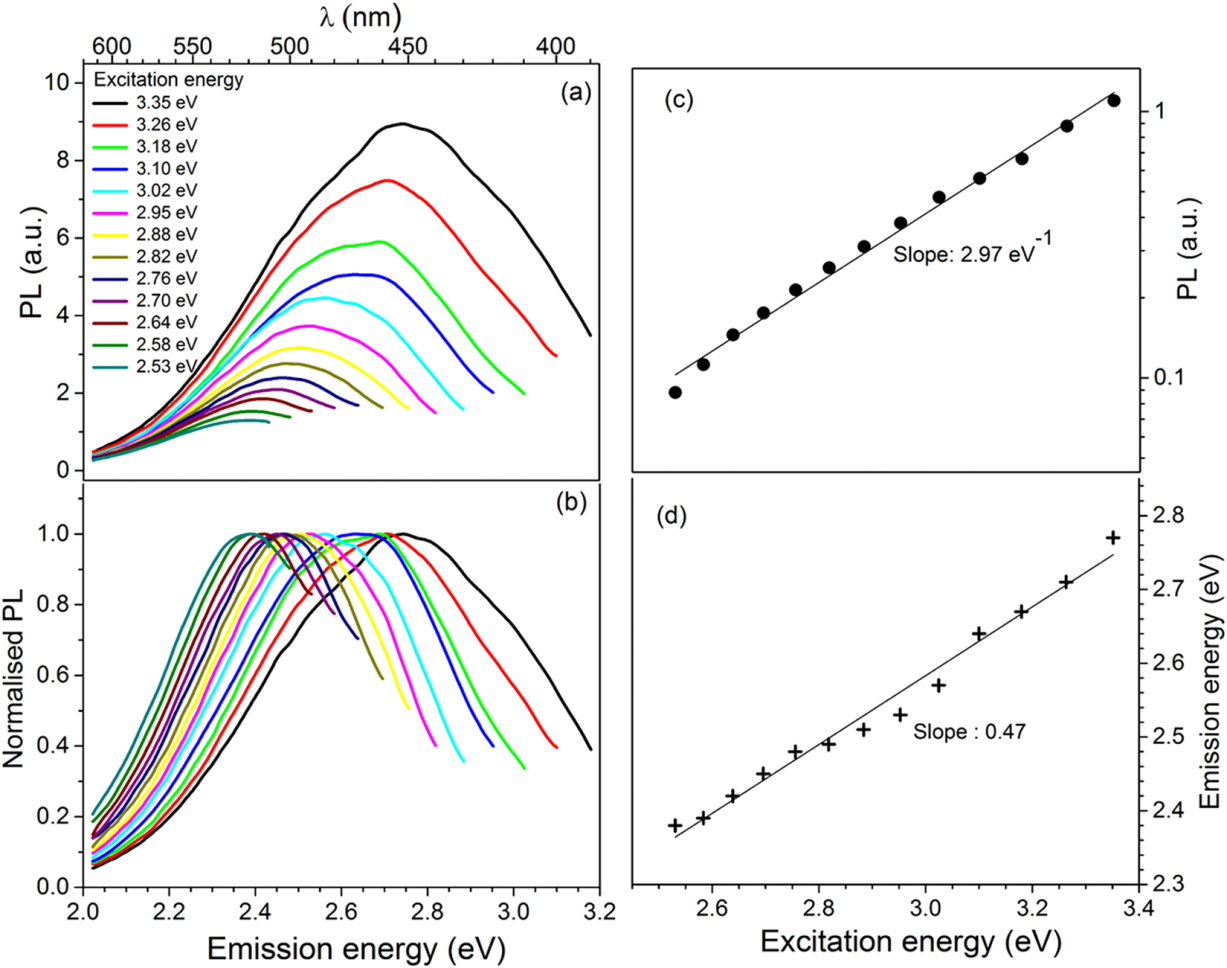
Dependence of PL emission on the excitation energy in sample R64. (**a**) Excitation-energy-dependent emission (EDE) spectra, (**b**) Normalized curves (by peak intensity) of the data in (**a**), (**c**) log_10_ of the PL peak area plotted as a function of excitation energy. The slope calculated using the natural logarithm of the PL data are also shown, (**d**) the emission peak of PL spectra plotted as a function of the corresponding excitation energy, demonstrating the excitation-energy-dependent emission (EDE) effect.

Our measurements on different kinds of feldspar show large spectral shifts in the PL emission (emission band: 2 0.1–2.8 eV) due to a change in the excitation energy. Figure 1 shows the example of excitation-energy-dependent PL emission spectra from sample R64 (Ca Feldspar, Table 1) at 295 K. The excitation energy was varied from ~3.35 eV (370 nm) to ~2.53 eV (490 nm) in the increments of 10 nm. Figure 1(a) shows the broad emission spectra obtained from the individual excitation energies. Figure 1(b) shows the normalised data from Fig. 1(a). Lowering the excitation energy causes a decrease in the emission peak energy and height as well as a narrowing of the emission spectrum, leading to a systematic decrease in the area under the PL emission curve. Figure 1(c) shows a plot of the log (PL area = net intensity) as a function of excitation energy; the data are well approximated by a single exponential with the slope of 2.97 eV^−1^. These data suggest that the excited levels giving rise to the PL distribution have a monotonic, exponential density distribution. Finally, it can be observed that the emission peak energy varies linearly with the excitation energy (Fig. 1(d)), with a slope of about 0.5.

To verify the universality of these results, we investigated this phenomenon in five other samples from the alkali and plagioclase series at 295 K. The detailed results following the same format as Fig. 1 are presented in the supplementary information (SI): Fig. SI 1 (R28 - K feldspar), Fig. SI 2 (R 47 - K feldspar), Fig. SI 3 (R57 – Na-K feldspar), Fig. SI 4 (R58- Na feldspar published in Prasad *et al*.^9^) and Fig. SI 5 (R65 – Na feldspar). We observe the same general behaviour as shown in Fig. 1 for all samples; the main difference is in the exponential constant and the energies spanning the EDE.

**Figure 2:**
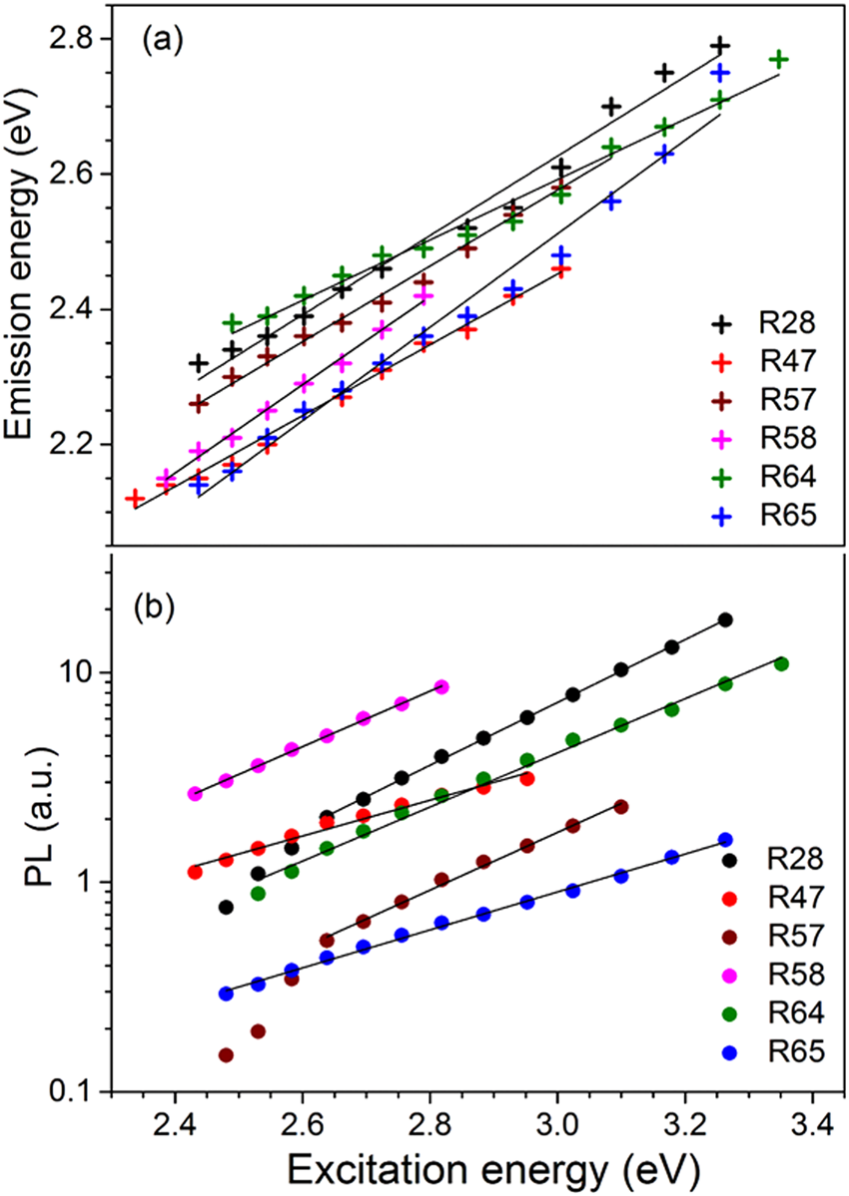
A summary of the excitation-energy-dependent emission (EDE) effect in all our samples. (**a**) PL emission peak vs. excitation energy. (**b**) log (PL area) vs. excitation energy.

A summary of the dependence of peak emission energy on excitation energy at 295 K is presented in Fig. 2(a). All samples show a linear dependence, with slope ranging from ~0.5–0.7 (see Table 2). These data suggest that there is a constant incremental change in the peak emission per unit change in the excitation energy. The net EDE was estimated as the difference in the peak positions of the emission spectra corresponding to the lowest and the highest excitation energy. On an average the EDE is about 90 nm and it varies from about 75 to 150 nm from sample to sample (see Table 2).

**Table 2:** EDE characteristics for different samples.

Sample name	Range of EDE peak emission@ 295 K [Range at 7 K]	Slope: Em_max_ vs. E @ 295 K [slope at 7 K]	Slope Ln(PL) vs. E @ 295 K [slope at 7 K] (eV^−1^)
R28	540–440 nm = 100 nm (~0.5 eV) **[550 to 450 nm = 100 nm]**	0.61 **[0.62]**	3.46 **[2.42]**
R47	590–500 nm = 85 nm (~0.4 eV)	0.56	1.97
R57	550–470 nm = 80 nm (0.4 eV)	0.59	3.46
R58	580–505 nm = 75 nm (0.3 eV) **[570–500 nm = 70 nm]**	0.70 **[0.72]**	3.05 **[3.08]**
R64	530–450 nm = 80 nm (0.4 eV)	0.47	2.97
R65	590–440 nm = 150 nm (0.7 eV)	0.72	2.09

Columns 2 shows an approximate range of EDE emission in different samples based on the lowest emission peak position (corresponding to the lowest excitation energy) and the highest emission peak position (corresponding to the highest excitation energy). Column 3 shows the slope of the linear correlation between the peak emission energy (Em_max_) and the excitation energy (E). Column 4 shows the Urbach slope (eV^−1^) derived using Equation 1, i.e. the slope of the ln(PL peak area) vs. excitation energy. All the estimates are based on measurements at 295 K, except those within square parenthesis which are measured at 7 K.

A summary of the log(PL area) vs. excitation energy at 295 K for all the samples is presented in Fig. 2(b). The samples R28 and R57 show a dominantly single exponential trend with a possible hint of a minor second exponential at excitation energy < 2.64 eV, whereas the samples R58, R64, R47 and R65 show a single exponential distribution of PL intensity as a function of the excitation energy (Fig. 2(b)). The exponential constant for the main component for each sample is summarised in Table 2; it is 3.46 eV^−1^ for R28 (Fig. SI 1(c)), 1.97 eV^−1^ for R47 (Fig. SI 2 (c)), 3.46 eV^−1^ for R57 (Fig. SI 3 (c)), 3.05 eV^−1^ for R58 (Fig. SI 4 (c)), and 2.09 eV^−1^ for R65 (Fig. SI 5 (c)).

**Figure 3:**
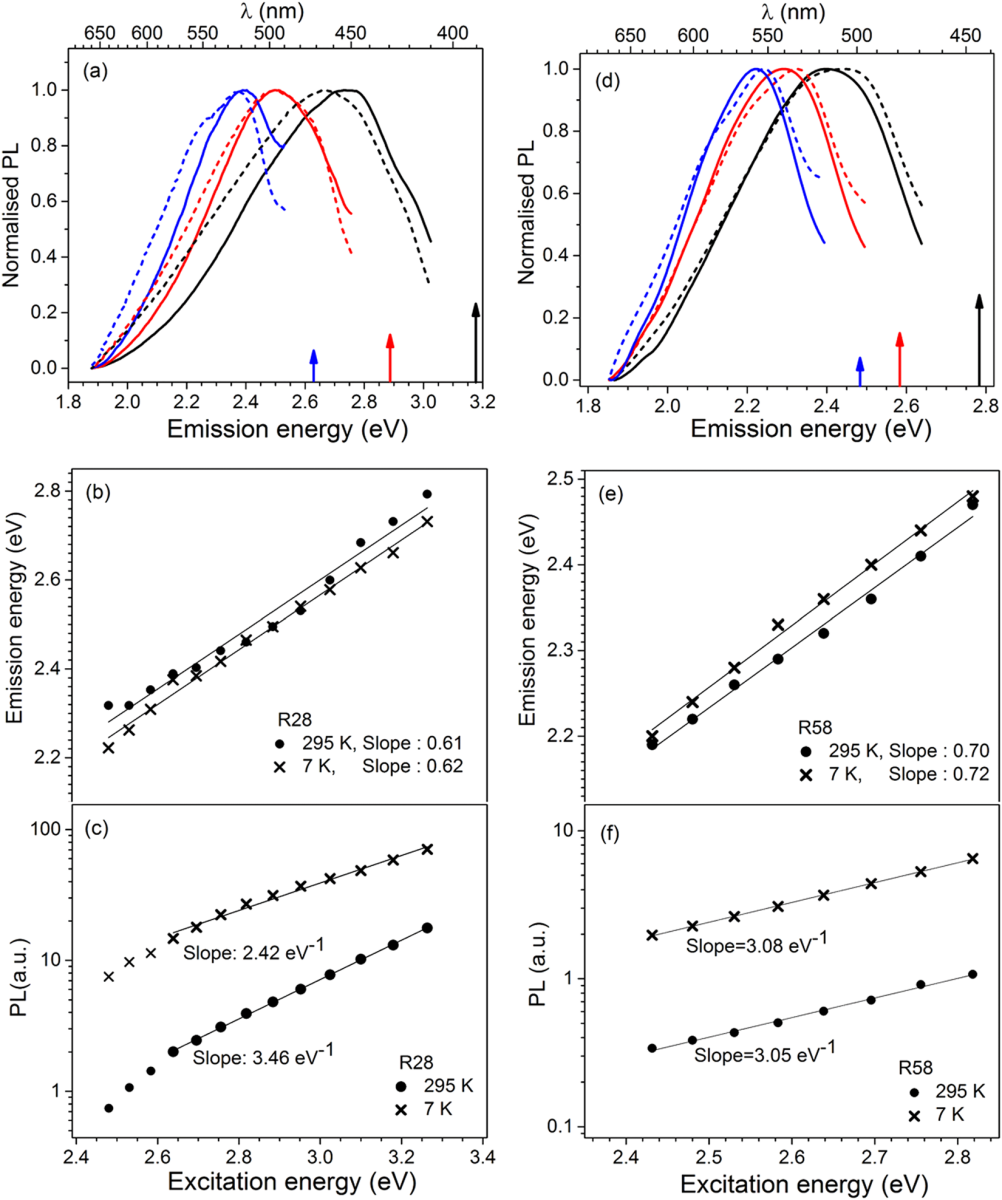
A comparison of 295 K and 7 K results on sample R28 (left column) and R58 (right column). (**a**) and (**d**) The emission spectra at 7 K or 295 K represented by dashed and solid curves, respectively. The emission spectra are obtained using excitation energy indicated by the same coloured arrows facing upwards. (**b**) and (**e**) Emission energy vs. excitation energy at 7 K or 295 K. (**c**) and (**f**) log (PL) vs excitation energy at 7 K or 295 K. The slopes are derived using the natural logarithm PL intensity data.

In order to understand the dynamics of the EDE and the possible role of the red edge effect^1^ identical measurements were made at 7 K on 2 samples with different compositions, R28 (K feldspar) and R58 (Na feldspar). Figure 3 presents the comparison of EDE at 7 K and 295 K in samples R28 (Fig. 3(a,b,c)) and R58 (Fig. 3(d,e,f)). The normalised emission spectra shown for three different excitation energies (marked with arrows) are shown in Fig. 3(a,d). The correlation between emission and excitation peak energy is shown in Fig. 3(b,e). The plots of log(PL) vs. excitation energy are shown in Fig. 3(c,f).

The emission spectrum for each excitation energy and its full width at half maximum (FWHM) is shown in the supplementary information (Fig. SI 6). FWHM was calculated using extrapolation where the high energy limb did not reach 50% of the peak height.

Some general inferences can be derived from these data:

- There is a manifold enhancement in the intensity for each emission at 7 K compared to 295 K (Fig. 3(c,f)). These data suggest a strong thermal quenching of the emission.
- FWHM is largely invariant as function of temperature (Fig. SI 6 (c) and (f)). In case of R28 there is no significant difference between FWHM of the emission spectrum at 7 K and 295 K; the average ΔFWHM (=FWHM_7K_ − FWHM_295K_) for emission spectra corresponding to different excitation energies is estimated to be 12 ± 42 meV (the uncertainty refers to 1 standard deviation). For R58, the there is no systematic trend in the FWHM difference as a function of excitation energy; the noise is partly due to the fact that FWHM is based on extrapolation in these data (Fig. SI 6 (d) and (e)). Nonetheless, the average ΔFWHM for different EDE in R58 is 26 ± 17 meV. This difference is of the order of about 5 ± 3% of the average FWHM for either 7 K or 295 K. These data demonstrate an insignificant broadening of emission at 7 K, thus, suggesting that REE mechanism is not so relevant for feldspar^1–8^.
- The slope of the emission peak energy vs. excitation energy is independent of measurement temperature (Fig. 3(b,e)). This observation is not surprising given that the 295 K and the 7 K spectra are largely identical in spectral width and form (see Fig. 3(a,d); Fig. SI 6 a,b,d,e).

Despite these general similarities, there is some sample dependent difference for the 7 K and 295 K data. The first main difference is in the exponential constant, i.e. the slope of ln(PL) vs. excitation energy (Fig. 3(c,f)). In case of R58 the exponential constant is essentially invariant at 295 K or 7 K (3.05 or 3.08 eV^−1^, respectively), whereas in case of R28, there is an apparent reduction in the slope from 3.46 eV^−1^ at 295 K to 2.42 eV^−1^ at 7 K. While in general, the peak shift and broadening are ambiguous and insignificant at 7 K, there appears to be a tendency in R28 that low energy limb is relatively enhanced at 7 K compared to 295 K (~5–10%); this effect is compensated by a slight relative suppression of the high energy limb (Fig. 3(a)). In R58, there is no such systematic effect (Fig. 3(d)); there are random cross-overs likely related to measurement noise.

### Time resolved luminescence (295 and 7 K)

In order to explore the dynamics of EDE in feldspar, we measured fluorescence and phosphorescence decay in R28.

**Figure 4:**
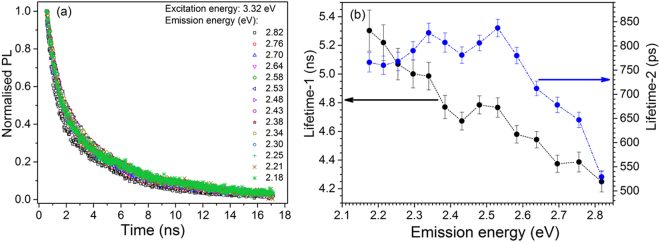
**(a) **Fluorescence decay of R28 at 295 K measured using 3.32 eV nanoLED excitation, and detection at different wavelengths spanning the PL emission peak in the increments of 10 nm. The data are analysed with a sum of two exponential functions. (**b**) Lifetimes derived from (**a**) as a function of emission energy (summarised in Table 3).

The fluorescence decay was measured by time-correlated single-photon counting technique using a ~3.32 eV pulsed nanoLED excitation at 295 K. The detection window was systematically varied from 440 nm (~2.82 eV) to 570 nm (~2.18 eV) in a step of 10 nm in different experiments. The data are shown in Fig. 4(a) and were fitted to a sum of two exponentials (fitting yield is presented in Table 3). Figure 4(b) shows the summary of the two lifetimes as a function of emission energy. Although the curves look very similar (see Fig. 4(a)), there appears to be a systematic decrease in the lifetime of the dominant slower component from 5.3 ns to 4.2 ns as the energy of the emission increases from 2.18 to 2.82 eV. The faster lifetime remains constant at about 800 ps from 2.2 to 2.6 eV and then decreases systematically with emission energy to a value of 500 ps at 2.8 eV.

**Table 3:** Summary of fitting parameters from the fluorescence and phosphorescence decay of sample R28, at room temperature or 7 K. Columns 2 and 3 present estimates from fluorescence decay (Figure 4). Columns 4-7 present estimates from phosphorescence decay measurements (Figure 5).

Emission energy (eV)	295 K				7 K	
	τ_1_ (ns)	τ_2_ (ps)	τ_1_ (ms)	τ_2_ (µs)	τ_1_ (ms)	τ_2_ (µs)
2.82	4.25 ± 0.07	529 ± 13	0.39 ± 0.01	45 ± 1	0.42 ± 0.01	71 ± 2
2.76	4.39 ± 0.07	647 ± 15				
2.70	4.37 ± 0.06	677 ± 16				
2.64	4.54 ± 0.06	711 ± 15				
2.58	4.58 ± 0.06	780 ± 17				
2.53	4.77 ± 0.06	837 ± 17	0.36 ± 0.01	52 ± 1	0.48 ± 0.02	67 ± 2
2.48	4.78 ± 0.06	806 ± 16				
2.43	4.67 ± 0.06	781 ± 17				
2.38	4.77 ± 0.08	807 ± 20	0.28 ± 0.01	54 ± 1	0.43 ± 0.01	54 ± 2
2.34	4.99 ± 0.10	827 ± 20				
2.30	5.00 ± 0.10	789 ± 19				
2.25	5.07 ± 0.11	768 ± 19				
2.21	5.22 ± 0.12	760 ± 19	0.29 ± 0.01	56 ± 1	0.36 ± 0.01	61 ± 1
2.18	5.30 ± 0.14	766 ± 20				

**Figure 5:**
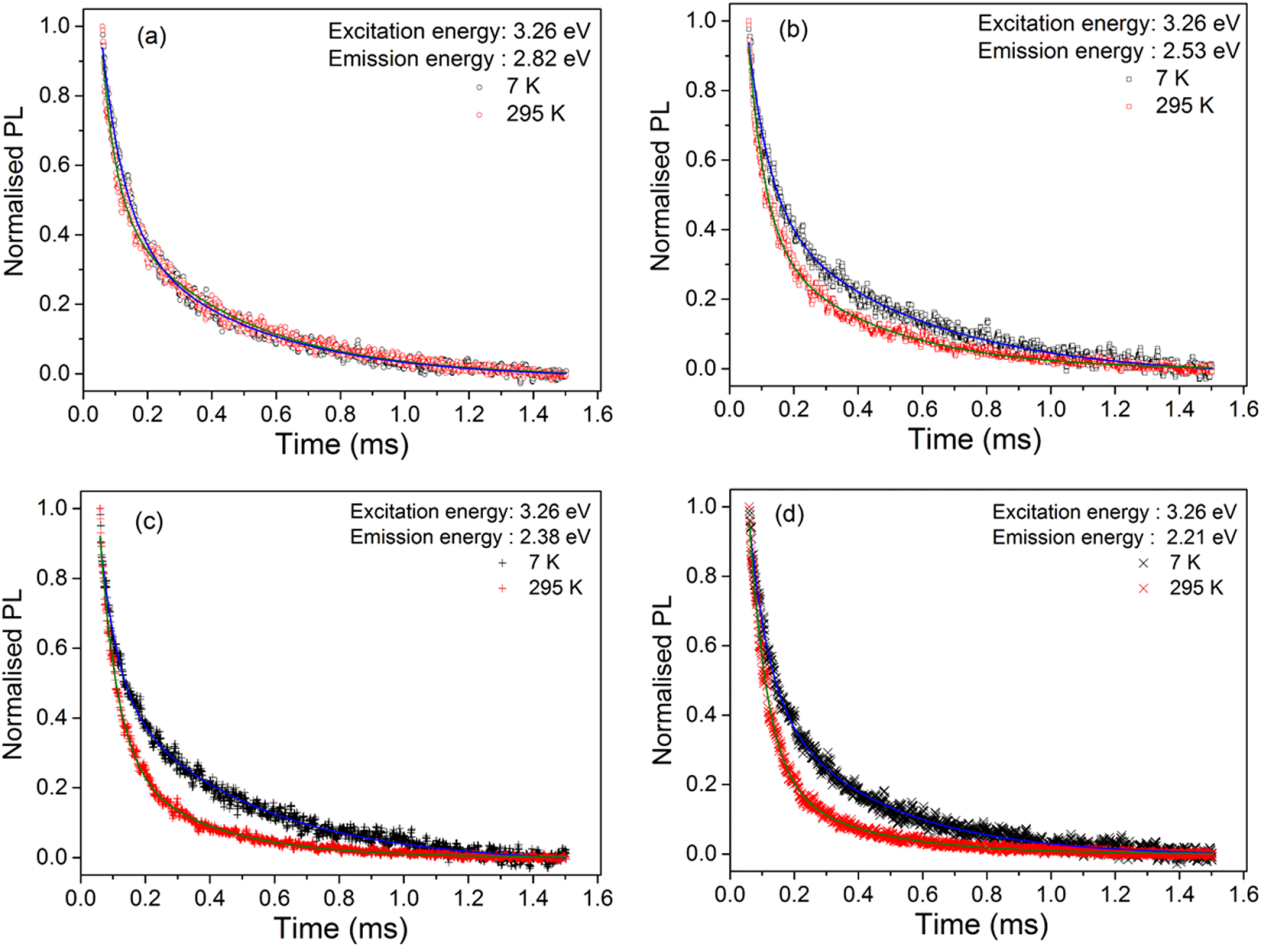
Phosphorescence decay measured at 7 K or 295 K using a 3.26 eV excitation selected from a pulsed Xe lamp. The phosphorescence data are plotted for 7 K and 295 K, using different emission windows (**a**) 2.82 eV (**b**) 2.53 eV (**c**) 2.38 eV (**d**) 2.21 eV. The data were fitted with sum of two exponentials and the lifetimes are summarised in Table 3.

Phosphorescence was measured at 7 K or 295 K, using a pulsed Xe lamp by selecting 3.26 eV from an excitation monochromator. The detection monitored for four different detection windows are shown in Fig. 5: (a) 2.82 eV, (b) 2.53 eV, (c) 2.38 eV and (d) 2.21 eV. The data were fitted to a linear combination of two exponential decays; the derived lifetimes are summarised in Table 3. Interestingly, the phosphorescence decay varies significantly at 295 K for different emission windows, with respect to that at 7 K. Specifically, there is an increase in the phosphorescence decay rate at 295 K with a decrease in the emission energy; the phosphorescence decay at 295 K is similar to that at 7 K for 2.82 eV emission, whereas it is faster than 7 K data for 2.38 and 2.21 eV emission. An intermediate scenario can be seen in the 2.53 eV emission.

In the next section we use these different results to derive a conceptual model for EDE in feldspar.

## Summary and Discussion

The orange-green emission in feldspar has been widely studied using cathodoluminescence (CL), radioluminescence (RL) and X-ray excited optical luminescence (XEOL)^9,31,32^. EDE covering this emission is prevalent in our feldspar samples comprising different compositions; a summary of the steady state PL results is given in Fig. 2 and Table 2. The excitation energy ranging from 2.38–3.26 eV leads to a PL emission in the 2.2–3.0 eV range; the EDE shift in our samples is as much as ~150 nm (Table 2; Figs 1, SI 1 to SI 6). In rigid, macro-sized (sub millimeter) materials, EDE is exhibited by disordered, amorphous materials and it is typically of the order of tens of nm (<50 nm): for example, low temperature glasses^1,4^, polymers^33–35^, amorphous semiconductors^36^, etc., due to the REE mechanism. Thus, the EDE magnitude observed in feldspar is unprecedented in rigid (solid) materials that are not based on nanostructures. What makes EDE in feldspar even more interesting is the fact that feldspar is crystalline, and not a disordered, amorphous medium which are known to exhibit REE. Given the fact that feldspar is the most abundant mineral on the Earth’s crust, it becomes imperative to seek explanation for the EDE in this material.

Feldspar is a wide bandgap (~7.7 eV) material, thus, the excitation energies used in this study are not sufficient to induce inter-band transitions. The origin of PL in our samples is, therefore, related to defect(s) energy level within the bandgap. The PL emission is rather broad (up to ~150 nm; Table 2) and the spectral width for a given excitation energy is very similar for measurements at 7 K or 295 K (Figs 3, SI 6). This suggests that the emissions dominantly arise from temperature independent, inhomogeneous broadening of the defect’s or the host’s energy levels^37–39^. The EDE may then arise from selective sub-sampling of the ensemble of such levels^1,36^.

The relationship between PL intensity and excitation energy is insightful. The integrated PL intensity increases exponentially as a function of excitation energy in all our samples (Fig. 2(b); this suggests that there exists an exponentially distributed continuum of excitation levels, which is available to the defect. However, the emission peak energy varies only linearly with the excitation energy with a slope of about ~0.6 on an average (Table 2), apparently supporting the case for a classic REE^6^. Nonetheless, the presence of a) a strong EDE spanning up to 150 nm, b) an exponential increase in PL intensity with excitation energy, c) and long-range order due to crystalline nature, and d) insignificant spectral broadening at 7 K, all suggest that REE model is not adequate to explain EDE in feldspar.

It is well known that crystalline semiconductors and insulators exhibit exponential optical absorption edges or Urbach edges arising from localised thermal and structural disorders in a crystal^40,41^. The defect levels near the band edge have been observed to result in a strong continuum of states in heavily doped semiconductors^42,43^. In particular, Coulomb impurities can cause a Gaussian probability distribution in deep tails and exponential distribution near the band edge where energy of localisation plays a dominant role in determination of the scale of the most probable potential fluctuation^41,42,44^. Specifically in feldspar, the band tail states and their active role in charge transport has been well established by spectroscopic and time resolved optically stimulated luminescence measurements^22,23^. Based on the data on R58 sample, Prasad *et al*.^9^ suggested that the exponential increase in PL intensity either maps the DOS of the sub-conduction band tail states, or it arises from a charge transfer band originating from defect clustering. In the current study, we have tested 5 more samples and we observe the exponential trend of PL intensity with excitation energy in all the samples (Figure 2(b)). Our data from the sample R28 is particularly notable as this sample was also used by Poolton *et al*.^22^ for direct mapping of the band tail states employing a combination of X-ray irradiation and excitation spectroscopy at 10 K as well as cryogenic thermoluminescence measurements. These authors derived a band tail width of ~0.4 eV. The slope of our exponential data for R28 at 7 K (similar to 10 K used by Poolton *et al*.^22^) is 2.42 eV^−1^; the inverse of this value gives the band tail width following Equation (1) of ~0.41 eV, which is in excellent agreement with the previous measurement by Poolton *et al*. using a different method^22^. These considerations suggest that the PL dependence on excitation energy is mapping the distribution of the band tail states in feldspar. Furthermore, the slope of ~0.6 of the emission vs. excitation energy in our data is similar to that observed in amorphous semiconductors^36^. This relationship has been explained by exponential density of localised states (i.e., band tail states) below the absorption edge, and subsequent thermalisation following multiple trapping^36,45^. Finally, the magnitude of EDE seems to be consistent with the width of the band tail states (Table 2) suggesting that the excited energy levels required to explain the large EDE in feldspar are available within the band tail states. Based on the above understanding, we propose the following phenomenological model for EDE in feldspar.

### EDE Mechanism

**Figure 6:**
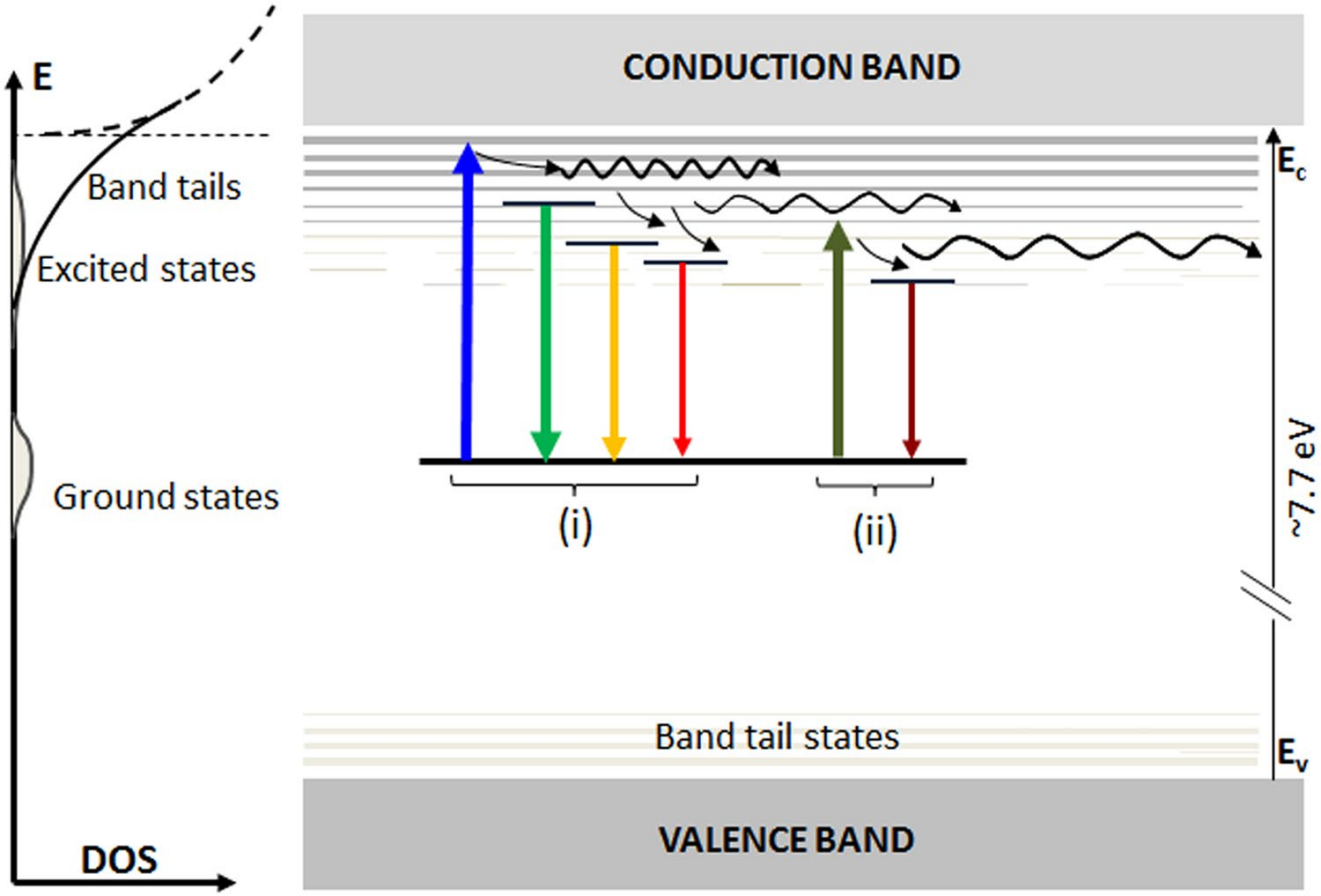
A conceptual model of the excitation-energy-dependent emission (EDE) effect in feldspar. E – energy, DOS- density of states, Upward arrow – optical excitation, downward arrow – radiative relaxation. Optical excitation leads to defect ionisation by transition of electrons to the band tail states. Subsequent thermalisation and hopping transport leads to the formation of a metastable excited state of the defect, followed by relaxation and PL emission. The wiggles in the band tail states represent the hopping process. A continuum of excitations and emission energies contribute to the excitation-dependent emission; cases (**i**) and (**ii**) are shown to represent relatively high and low energy cases, respectively. Importantly, a decrease in the excitation (case (**ii**)) leads to a simultaneous decrease in the emission energy, and any PL produced using a certain excitation energy is a subset of the PL produced from a relatively higher energy. Localised recombination is expected to dominate for the low energy band tail states.

Our model as shown in Fig. 6 proposes that EDE occurs through a following sequence of steps:

1. Photo-ionisation of a deep defect leading to a transition of electrons from the ground state (E_g_) of the defect to the band tail states. The DOS of the band tail states follows the well-known relation (Equation 1). Further, it is assumed that ground states of the defect are distributed (Poisson or Gaussian) and lie far below the band tail states (e.g. at > 4E_b_), whereas the excited levels (E_ex_) have a distribution embedded within dense, high energy band tail states.
2. Hopping transport and thermalisation of free electrons within the band tail states. This relaxation process eventually leads to some retrapping into the excited energy levels of the defect which are embedded within the band tail states. The hopping length and thermal activation of hopping are dependent on the DOS, which is a function of the band tail energy; in general, larger for lower energy band tail states and smaller for the higher energy states.
3. Radiative relaxation of the excited state of the defect resulting in photon emission in the 2.2–2.8 eV range.

Thus, an EDE is produced since there is a continuum of the excitation and emission energies available within the band tail states, and the PL emission primarily occurs from states that are below the excitation energy due to thermalisation (Fig. 6). The overall peak shape of the emission likely arises due a quasi-equilibrium distribution of electron occupancy in the band tails resulting from a combination of i) the exponential DOS of the band tails, ii) the distribution of the zero phonon transition levels of the defect, and iii) the temperature dependent Boltzmann’s distribution.

This model potentially also explains the deviation from the exponential trend at low excitation energies observed in some samples (Fig. 2(b)). At low energies, the low DOS of the band tails is comparable with the DOS of the excited states (Fig. 6); this is especially true for samples with fewer band tail states. Thus, photoionisation leading to free electrons in the band tail states will compete with intra-defect excitation. The latter will not significantly contribute to EDE because of lack of redistribution of electrons to different energy levels necessary for a broad EDE. Excitation photons lost to intra-defect transition will thus lead to a decreased efficiency of EDE. Other factors for deviation at low energies may be presence of a mixed feldspar phase; however, we do not see any direct correlation between bulk feldspar composition and the deviation from exponential behaviour at low energies (Table 1, Fig. 2). For example, R65 has 27% K and 73% Na, but it shows a linear trend, whereas R28 is 95% K and 5% Na, but it shows a deviation at low excitation energies. Finally, it is also possible that the density of localised states assume a Gaussian distribution far from the band edges^41,46^. These different explanations should be explored in future work. A linear change in the emission peak with excitation energy (Fig. 2(a)) with a slope of ~0.6 is qualitatively consistent with the predictions of the band tail states model^36^.

The fact that there is undetectable (in R28) or insignificant (in R58 at 2σ) spectral broadening at 7 K suggests that the classic REE mechanism does not play an important role in feldspar. This is not unexpected since feldspar is a crystalline (ordered), rigid material. REE is typically important in rigid medium with high disorder (e.g. amorphous) characterised by many microscopic configurations that are structurally and energetically different^1^. Furthermore, in R28, there is a systematic tendency for a slight red shift at 7 K (Fig. 3(a)); this is contrary to the REE where a blue shift is expected at low temperatures^34^. In the band tail model, proposed here, the red shift at low temperatures can arise from Boltzmann’s distribution, i.e. electron occupancy will be biased towards the lower energy band tail states at 7 K compared to that at 295 K.

The reduction in the PL intensity at 295 K in comparison to 7 K is likely a combination of thermal quenching of the luminescence emission due to phonon relaxation at the defect site. Additionally, it is likely that a loss of electrons from the band tail states may occur to other trapping sites in the crystal due to enhanced diffusion at high temperatures^19,22,23^. Interestingly, in R58 the exponential constant ln(PL) vs. excitation energy plot is the same at 7 K and 295 K (Fig. 3(f)), but it is different for R28 (Fig. 3(c)). One may be tempted to think that the different slopes may be due to the temperature dependence of the Urbach slope and the energy gap due to absorption sidebands created by nonlinear electron-phonon interaction^47^. However this explanation does not account for why the effect is observed in R28 and not R58 even though they are both feldspar lattice. One explanation for the apparent change in the Urbach slope may be the likely energy dependence of the retrapping process. For low excitation energies, there will be a relatively higher rate for localised retrapping at 7 K compared to that at 295 K; this is because at higher temperatures hopping becomes active and the electrons may be lost to higher energy states or non-radiative routes (e.g., other defects). However, for higher excitation energies, which access higher DOS, the retrapping rate will not greatly alter at 7 K (compared to 295 K); this is because thermal activation is much lower in shallow (high energy) band tails states due to a larger wave-function overlap, compared to the deeper states^20,23,27^. Thus at 7 K, a systematic relative increase in retrapping efficiency from the lower band tail states (corresponding to smaller excitation energies leading to greater PL since same traps are available to be excited multiple times) explain both a decrease in Urbach slope as well as the red shift observed in R28. However, this effect will be dampened if there is an absolute increase in the overall density of band tail states (integral of Equation 1), such that hopping is relevant for deep band tail states even at 7 K, which may be the case of R58. Thus, sample to sample dependence in the absolute density of band tail states may control the temperature dependent Urbach slope as well as the red shift at low temperatures.

Time-resolved luminescence can give further information on relaxation and transport time scales in feldspar^23,27^. Our fluorescence lifetime results are essentially invariant with the emission energy, likely representing the fundamental transition (possibly phonon mediated) from the excited to the ground state of the defect (Fig. 4(a)). The minor (1 ns) but systematic variation in the lifetime from the high to the low emission energy is interesting; it possibly reflects a relationship between the fundamental lifetime and the corresponding transition energy (E_ex_ − E_g_), possibly due to interaction between the excited state and the band tail states. It has been shown that the Band tail model predicts a strong energy dependence of the radiative recombination lifetime^48^. Alternatively, one may consider a partial REE mechanism. It may be argued that disordered band tail states act like a glassy matrix around the defect; thus a limited dipole reorganisation in a rigid matrix could give rise to the decrease in the lifetime for low energy band tail states; this effect is similar to the classic red edge effect observed in polar solvents^1^.

The phosphorescence data show lifetime components in the microsecond and millisecond time scales (Fig. 5). These lifetimes can be attributed to the hopping mechanism suggested in our EDE model (Fig. 6). As discussed earlier, we expect that sample temperature should not affect the transport (hopping) times for the high energy band tail states, since a large DOS leads to large wave-function overlap. Our data confirm this expectation; there is no significant difference between 7 K and 295 K phosphorescence decay in case of the 2.82 eV emission (Fig. 5(a). On the other hand, for low energy emissions thermally assisted hopping and loss to other routes should be important when there is small wave-function overlap between the states. As expected, there is much faster decay of the PL as the temperature is raised from 7 K to 295 K for the 2.28 and 2.21 eV emissions (Fig. 5(c,d) confirming that the lower band tail states empty faster due to the thermal effect. The 2.53 eV emission shows intermediate emptying rate (Fig. 5(b) at room temperature.

### Luminescent defect

It is generally assumed that the orange-green band in feldspar, which is a subset of our EDE, is due to Mn^2+^ substituting for the Ca sites^49^. The Mn content in our samples in shown in Table 1. We did not observe any correlation between the emission intensity and the Mn content in our six samples. Furthermore, the typical orange emission in Mn^2+^ takes place via the d^5^ spin-forbidden transition (^4^T_1_ (G) → ^6^A_1_(S)); thus, the relaxation lifetimes are expected to be in millisecond range^9,49,50^. Even in disordered glasses, Mn^2+^ lifetimes have been observed to be on the milliseconds time scales^50^. Given these considerations, our lifetime data in the picosecond to microsecond range, suggest that a simple Mn^2+^ emission model cannot be applied to EDE in feldspar.

There could be other possible candidates for the luminescent defect. It has been demonstrated that surface states may play an important role in the EDE in carbon based quantum dots^5,51^. Fitting *et al*.^52^ demonstrated the appearance of the green-yellow band in silicon nanoclusters (~2 nm diameter); they attributed these to the oxygen deficiency centers^52^. It is not inconceivable that the EDE may be arising from the alteration sites in Si tetrahedra, which are possibility more dominant at the crystal surfaces^53^. The determination of the exact defect states requires further investigations.

The defect and band tail state interaction model proposed here may be worth considering other solid materials exhibiting the EDE. For example, band tail states are also known in semiconductor nanomaterials of different compositions^54–56^. The band tail model proposed here contributes further to the debate on the origin of the EDE in rigid materials, and hopefully inspires further investigations of this effect in wide bandgap materials.

## Conclusion and Outlook

We demonstrate here a strong Excitation-energy-Dependent photoluminescence Emission (EDE) in a crystalline, naturally occurring, wide bandgap aluminosilicate (feldspar), which is the most dominant mineral in the Earth’s crust (~60% of terrestrial rocks). We conclude that this effect arises from interaction between a deep defect and the sub-conduction-edge band tail states. We also conclude that the orange-green emission does not arise from Mn^2+^ as is commonly believed. Based on our new model of the excitation-dependent emission, we propose a simple, robust method for measurement of band tail width in feldspar; as issue of immense relevance for its application in geochronometry and thermochronometry. The model proposed here has implications for understanding EDE in other rigid systems and carbon based nanostructures.

## Samples and experimental details

Six museum and sedimentary feldspar samples (alkali and plagioclase feldspars) were used in this study. The concentrations of K-feldspar (KF), Na-feldspar (NaF) and Ca-feldspar (CaF) were estimated using X-ray fluorescence spectroscopy (XRF) attachment in the Risø TL/OSL reader, and the Mn concentration was measured using the thermo X-series II – Quadrupole inductively coupled plasma mass spectrometry (ICP-MS) (see Table 1). The grain size used in the present studies was in the range of 90–180 µm, whereas R28 was a single crystal of 2 × 2 × 2 mm size. R28 has been previously characterised extensively for its optical properties^57–59^. Poolton *et al*.^22^ used this sample for measurement of band tail states; hence, it serves as a reference sample for our study. Sample R58 is the same sample as that reported earlier^9^; however, note that the K, Na and Ca concentrations presented here using the XRF are slightly different from those measured using the ICP-MS in the previous study^9^. This difference likely represents a sub-sample variability in the major cation concentrations. Sample R65 is a Na feldspar standard from National Institute of Standards and Technology, USA (NIST sample code: SRM 99b).

The excitation-energy-dependent emission (EDE) measurements at room temperature (295 K) and at cryogenic temperature (7 K) were carried out using Risø station for CryOgenic LUminescence Research (COLUR) at DTU Nutech, Technical University of Denmark, Risø campus, Roskilde-4000, Denmark. This facility consists of a Horiba Fluorolog-3 spectrofluorometer, a He closed loop cryostat and multiple excitation sources and detectors. Sample temperature can be controlled between 7–300 K, which is mounted to the cold figure of cryostat under vacuum (2.5 × 10^−5^ mb). A 450 W Xenon CW lamp with a wavelength range of approximately 370–520 nm was used for excitation, with the specific wavelengths selected using a double grating Cherny-Turner excitation monochromator. Emission spectra from 385–650 nm were detected using a similar monochromator in front of a photomultiplier tube. All the spectra were corrected for the instrument response and photon flux.

The photoluminescence lifetime of the order of millisecond - microsecond was measured using a pulsed Xe lamp and a multi-channel analyser. The specific wavelengths of the excitation pulse were selected with an excitation monochromator, while emission wavelength was selected with the emission monochromator in front of the PMT detector. The integration time for the data collection was 100 ms, and the initial delay of the system was 0.05 ms; all the measurements used flash counts of 100 to improve counting statistics. The photoluminescence lifetime in the order of nanosecond was measured with the time-correlated single photon counting (TCSPC) system; excitation source was a nanoLED of wavelength 374 nm (~3.32 eV) operated at 1 MHz repetition rate.

## Electronic supplementary material

Supplementary information
