# Supplementary material for: Breakdown of Kasha’s Rule in a Ubiquitous, Naturally Occurring, Wide Bandgap Aluminosilicate (Feldspar)
Source: Sci Rep. 2018 Jan 16;8:810. doi: 10.1038/s41598-017-17466-z (PMC5770446; doi:10.1038/s41598-017-17466-z)
Supplement: Supplementary file 1 — Supplementary information [file 41598_2017_17466_MOESM1_ESM.pdf]

Breakdown of Kasha's Rule in a Ubiquitous, Naturally Occurring, Wide Bandgap Aluminosilicate (Feldspar)

Amit Kumar Prasad[1,2]* & Mayank Jain[1]

[1]*Center for Nuclear Technologies, Technical University of Denmark*

DTU Risø Campus, Roskilde-4000, Denmark

[2]*Present address: Schulich Faculty of Chemistry and Solid State Institute*

Technion – Israel Institute of Technology, Haifa, 32000, Israel

(* Corresponding author: amitphotonics@gmail.com)

Supplementary information (SI)

Contents		Page number
Figure SI-1	EDE characteristics of sample R28	2
Figure SI-2	EDE characteristics of sample R47	3
Figure SI-3	EDE characteristics of sample R57	4
Figure SI-4	EDE characteristics of sample R58	5
Figure SI-5	EDE characteristics of sample R65	6
Figure SI-6	EDE characteristics at 7 K or 295 K for R28 and R58	7

Figure SI-1

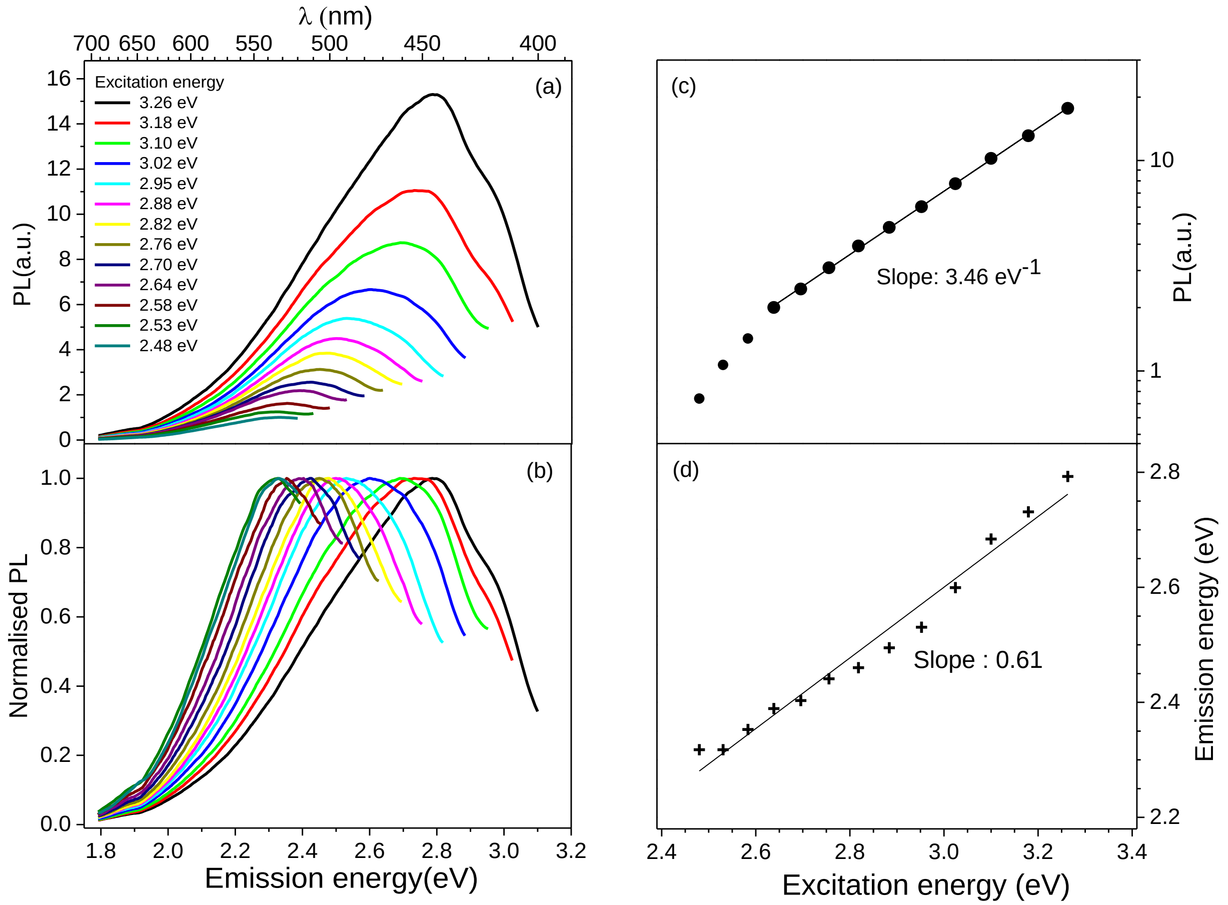

Figure SI-1. Dependence of PL emission on the excitation energy in sample **R28**.

(a) Excitation-energy-dependent emission (EDE) spectra, (b) Normalized curves (by peak intensity) of the data in (a), (c) log10 of the PL peak area plotted as a function of excitation energy. The slope calculated using the natural logarithm of the PL data are also shown, (d) the emission peak of PL spectra plotted as a function of the corresponding excitation energy, demonstrating the excitation-energy-dependent emission (EDE) effect.

Figure SI-2

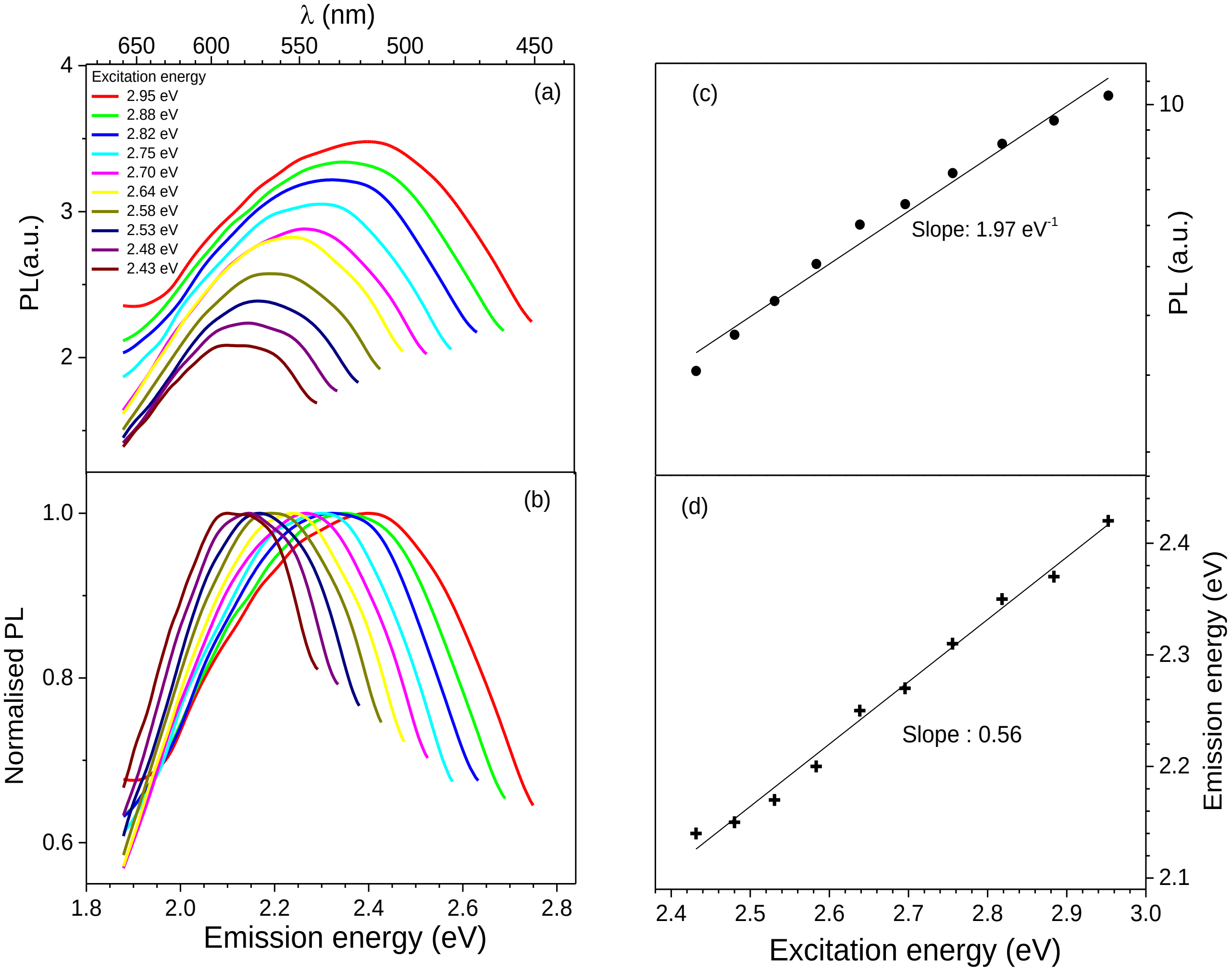

Figure SI-2. Dependence of PL emission on the excitation energy in sample **R47**.

(a) Excitation-energy-dependent emission (EDE) spectra, (b) Normalized curves (by peak intensity) of the data in (a), (c) log10 of the PL peak area plotted as a function of excitation energy. The slope calculated using the natural logarithm of the PL data are also shown, (d) the emission peak of PL spectra plotted as a function of the corresponding excitation energy, demonstrating the excitation-energy-dependent emission (EDE) effect.

Figure SI-3

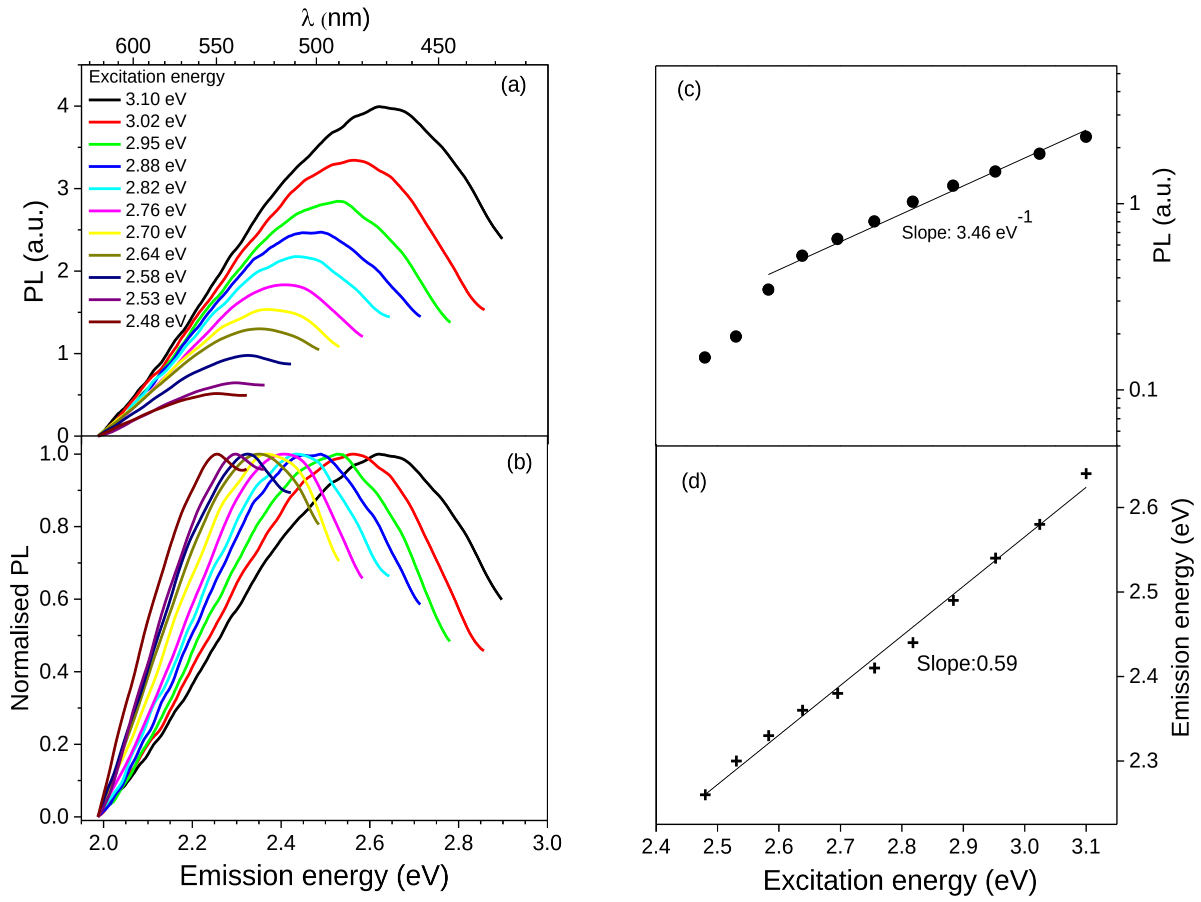

Figure SI-3. Dependence of PL emission on the excitation energy in sample **R57**.

(a) Excitation-energy-dependent emission (EDE) spectra, (b) Normalized curves (by peak intensity) of the data in (a), (c) log10 of the PL peak area plotted as a function of excitation energy. The slope calculated using the natural logarithm of the PL data are also shown, (d) the emission peak of PL spectra plotted as a function of the corresponding excitation energy, demonstrating the excitation-energy-dependent emission (EDE) effect.

Figure SI-4

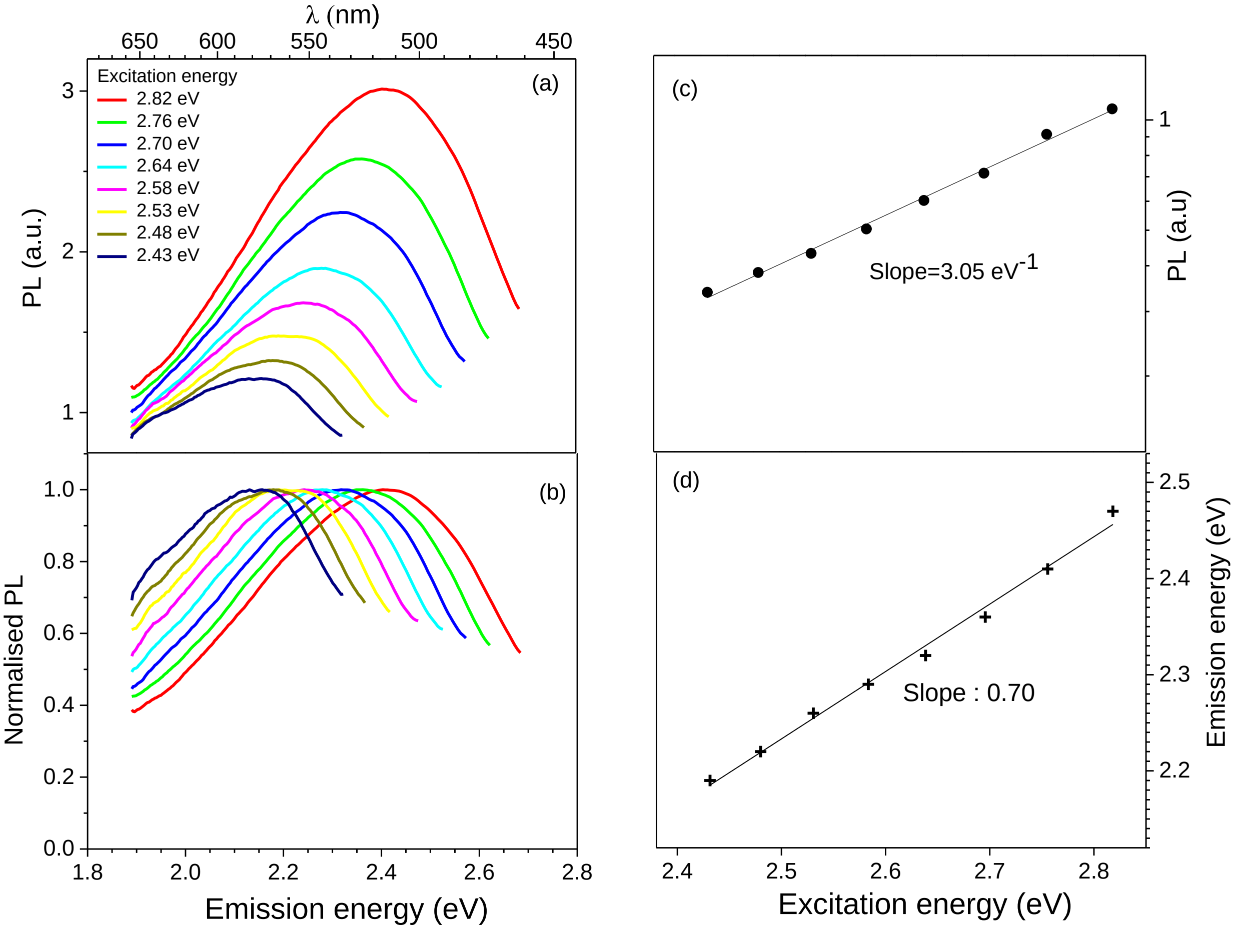

Figure SI-4. Dependence of PL emission on the excitation energy in sample **R58**.

(a) Excitation-energy-dependent emission (EDE) spectra, (b) Normalized curves (by peak intensity) of the data in (a), (c) log10 of the PL peak area plotted as a function of excitation energy. The slope calculated using the natural logarithm of the PL data are also shown, (d) the emission peak of PL spectra plotted as a function of the corresponding excitation energy, demonstrating the excitation-energy-dependent emission (EDE) effect.

Figure SI-5

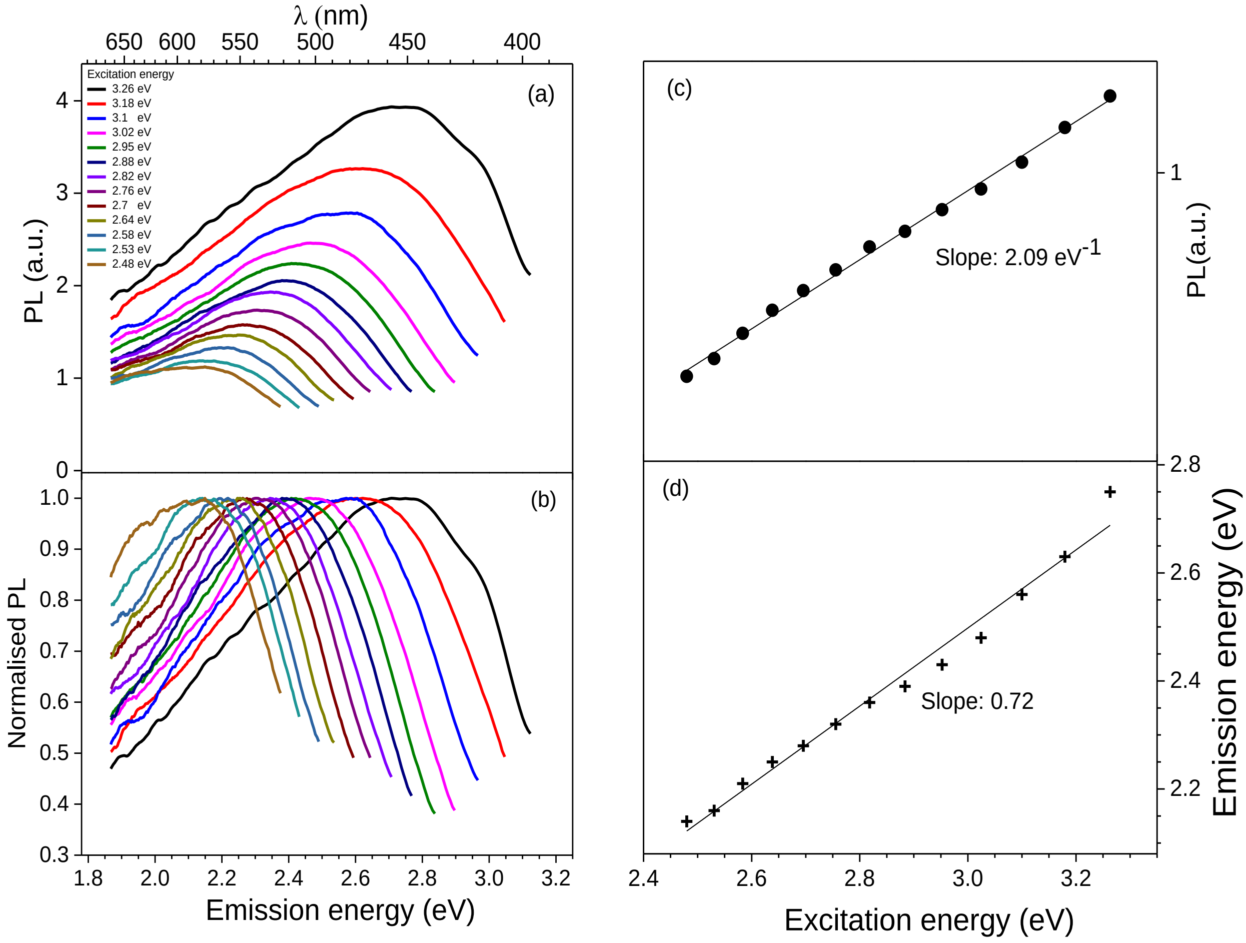

Figure SI-5. Dependence of PL emission on the excitation energy in sample **R65**.

(a) Excitation-energy-dependent emission (EDE) spectra, (b) Normalized curves (by peak intensity) of the data in (a), (c) log10 of the PL peak area plotted as a function of excitation energy. The slope calculated using the natural logarithm of the PL data are also shown, (d) the emission peak of PL spectra plotted as a function of the corresponding excitation energy, demonstrating the excitation-energy-dependent emission (EDE) effect.

Figure SI-6

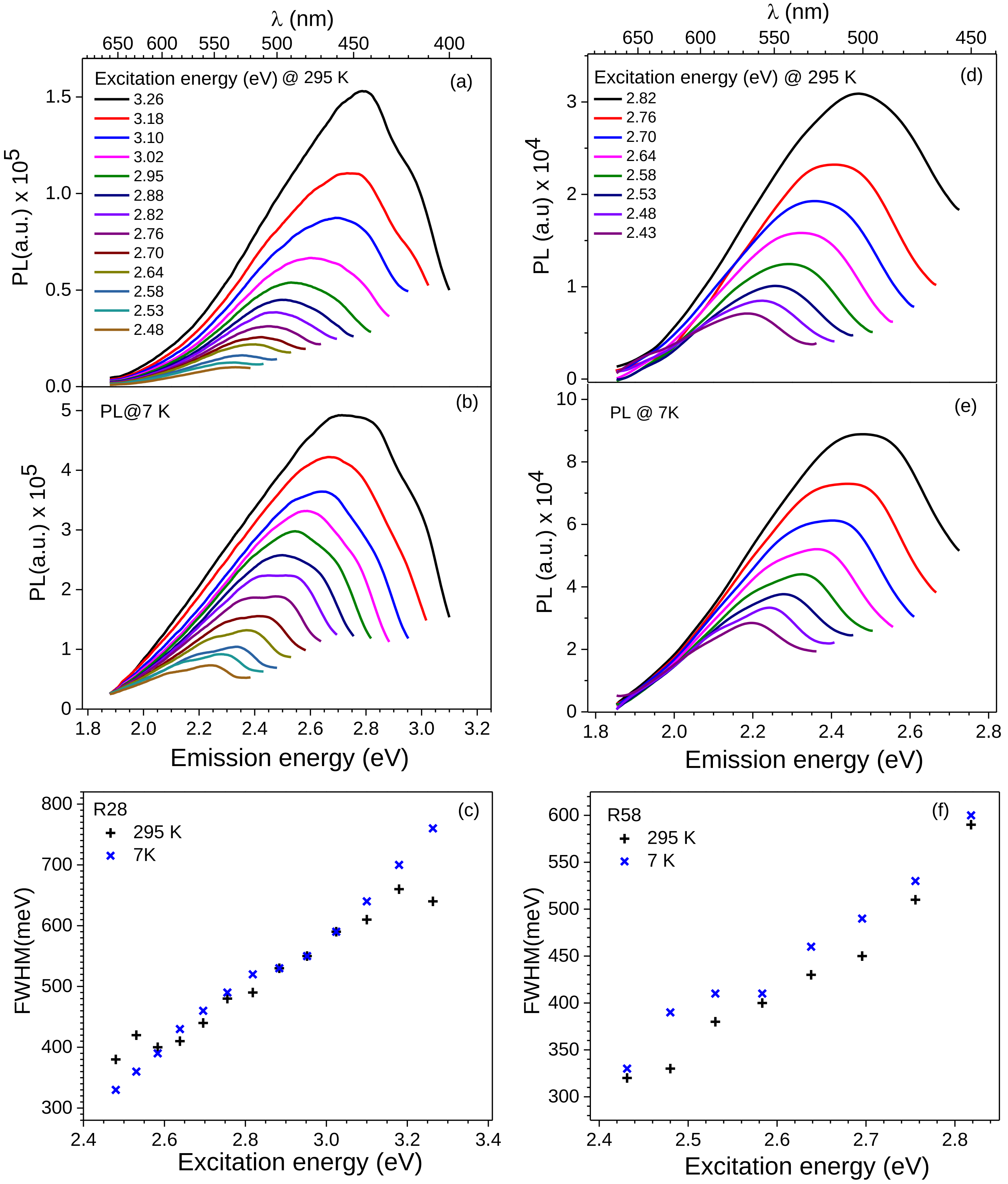

Figure SI-6: EDE at 7 K or 295 K. The left column shows results from R28 whereas the right column shows results from R58. (a) and (d): EDE at 295 K. (b) and (e) EDE at 7 K. (c) and (f): Full width at half maximum (FWHM) derived from the data shown above for 7 K or 295 K.
